# Phosphonium-Based Polyelectrolytes: Preparation, Properties, and Usage in Lithium-Ion Batteries

**DOI:** 10.3390/polym15132920

**Published:** 2023-06-30

**Authors:** Muhammad Syukri Mohamad Misenan, Rolf Hempelmann, Markus Gallei, Tarik Eren

**Affiliations:** 1 Department of Chemistry, College of Arts and Science, Davutpasa Campus, Yildiz Technical University, 34220 Istanbul, Turkey; syukrimisenan@gmail.com; 2 Transfercentre Sustainable Electrochemistry, Saarland University and KIST Europe, 66123 Saarbrücken, Germany; 3 Polymer Chemistry, Saarland University, Campus C4 2, 66123 Saarbrücken, Germany; 4 Saarene—Saarland Center for Energy Materials and Sustainability, Campus C4 2, 66123 Saarbrücken, Germany

**Keywords:** polymeric ionic liquid, phosphonium, polymer electrolyte, energy storage, lithium-ion batteries

## Abstract

Phosphorous is an essential element for the life of organisms, and phosphorus-based compounds have many uses in industry, such as flame retardancy reagents, ingredients in fertilizers, pyrotechnics, etc. Ionic liquids are salts with melting points lower than the boiling point of water. The term “polymerized ionic liquids” (PILs) refers to a class of polyelectrolytes that contain an ionic liquid (IL) species in each monomer repeating unit and are connected by a polymeric backbone to form macromolecular structures. PILs provide a new class of polymeric materials by combining some of the distinctive qualities of ILs in the polymer chain. Ionic liquids have been identified as attractive prospects for a variety of applications due to the high stability (thermal, chemical, and electrochemical) and high mobility of their ions, but their practical applicability is constrained because they lack the benefits of both liquids and solids, suffering from both leakage issues and excessive viscosity. PILs are garnering for developing non-volatile and non-flammable solid electrolytes. In this paper, we provide a brief review of phosphonium-based PILs, including their synthesis route, properties, advantages and drawbacks, and the comparison between nitrogen-based and phosphonium-based PILs. As phosphonium PILs can be used as polymer electrolytes in lithium-ion battery (LIB) applications, the conductivity and the thermo-mechanical properties are the most important features for this polymer electrolyte system. The chemical structure of phosphonium-based PILs that was reported in previous literature has been reviewed and summarized in this article. Generally, the phosphonium PILs that have more flexible backbones exhibit better conductivity values compared to the PILs that consist of a rigid backbone. At the end of this section, future directions for research regarding PILs are discussed, including the use of recyclable phosphorus from waste.

## 1. Introduction

Numerous energy storage technologies have been developed as a result of the rising demand for high energy density storage [1,2]. Technology for batteries has recently advanced quickly, starting with lead-acid batteries and moving on to nickel-cadmium, nickel-metal hydride, and lithium-ion batteries (LIBs) [3]. The market for LIBs is anticipated to increase to USD 139.36 billion by 2026 from USD 29.86 billion in 2017 [4]. Since Sony commercialized them in 1991, LIBs have dominated the portable electronics industry and have enormous potential for use in numerous applications like portable gadgets and electric vehicles (EVs) [5]. The usefulness of LIBs in daily life led to their inclusion in the 2019 Nobel Prize in Chemistry [6].

The LIB’s energy density is normally enhanced by high-voltage active cathode and anode materials (electrodes). Another essential component of LIBs is the electrolyte. It needs to guarantee quick ion transport and enough chemical and electrochemical stability, as well as overcome any safety concerns brought on by thermal instability, flammability, and leakage potential [7]. Unfortunately, the decomposition of the electrolyte at more than 4.2 V vs. Li/Li^+^ is one of the major issues with high-voltage cathodes in LIBs [8]. Traditional organic liquid electrolytes have significant safety issues since they are flammable and caustic [9,10,11,12]. Additionally, a significant barrier to the commercialization and scaling up of lithium-metal batteries is the liquid electrolyte’s inability to prevent the production and growth of lithium dendrites, which can result in battery shortcuts and explosions. The stability of the substance that replaces today’s electrolyte can therefore be seen as a crucial electrochemical characteristic in the implementation of next-generation devices that hold larger cell potentials. Li-ion batteries face new challenges as a result of the rapid development of electric vehicles and power plants in terms of safety and energy density (LIBs) [13].

Replacing conventional liquid electrolytes with other types of electrolytes is expected to solve battery safety issues, lessen side reactions at the electrolyte/electrode contact, enable the use of lithium metal anodes, and extend the lifetime of the battery. Neoteric electrolytes such as ionic liquids (ILs) and polymeric ionic liquids (PILs) have great potential for use in battery design. Due to the urgent safety issue regarding solvents as industry seeks batteries with smaller volume and higher power density, several academics are investigating ILs and PILs as electrolytes as opposed to traditionally utilized solvent-based electrolytes [14].

ILs were described as salts with melting points lower than the boiling point of water [15]. High thermal, chemical, and electrochemical stability and high ion concentration are typical characteristics of ILs [16,17] Wide liquid-phase windows and protection from fire dangers are two significant advantages that ILs have over solvent-based electrolytes due to their great stability and non-volatility. When using ILs as electrolytes, large ion densities also impart high ion conductivities, which is crucial in LIBs application [14]. On the other hand, the usually high viscosity is a problem.

Nonetheless, as it is still in the liquid state, uncontrolled dendritic lithium production can still occur, which would raise safety concerns for ILs. To prevent an electrical short-cut produced by dendrites penetrating the electrolytes, a straightforward approach is to give them mechanical integrity [17]. Thus, PILs (macromolecular forms of polymerizable ILs) have received increasing attention because they typically exhibit higher mechanical integrity than their IL analogues. The distribution of ILs in the matrix can change over time and can also leach out of the matrix. For this reason, PILs might provide better matrix compatibility in the long run.

**Figure 1:**
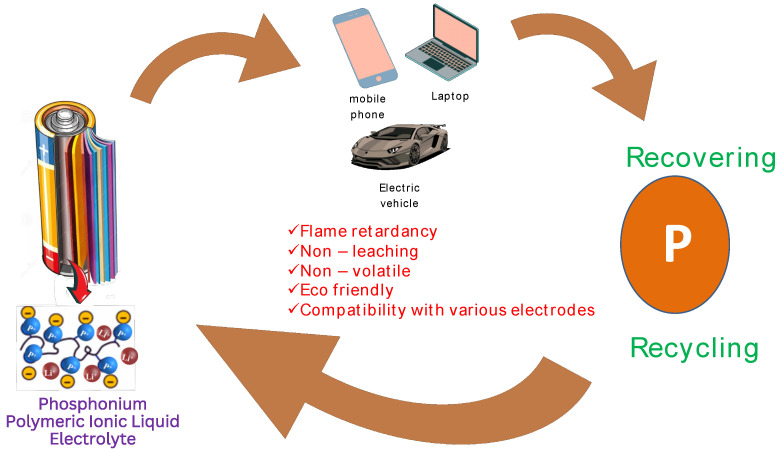
The use of phosphonium ionic liquids in lithium batteries and their advantages.

Polymeric ionic liquids (PILs) are a type of polyelectrolyte that feature an ionic liquid (IL) moiety in each monomer repeating unit, which is linked together by a polymeric backbone to form a macromolecular structure. PILs combine some of the unique properties of ILs in the polymer chain, resulting in a new class of polymeric materials. Rapid advances in the chemistry and physics of polyelectrolytes have resulted in the development of unique and versatile polyelectrolytes that are important for basic research and provide materials for new solutions in a variety of systems. Figure 1 shows the properties of phosphonium-based polymeric ionic liquids and their use in lithium-ion batteries.

**Figure 2:**
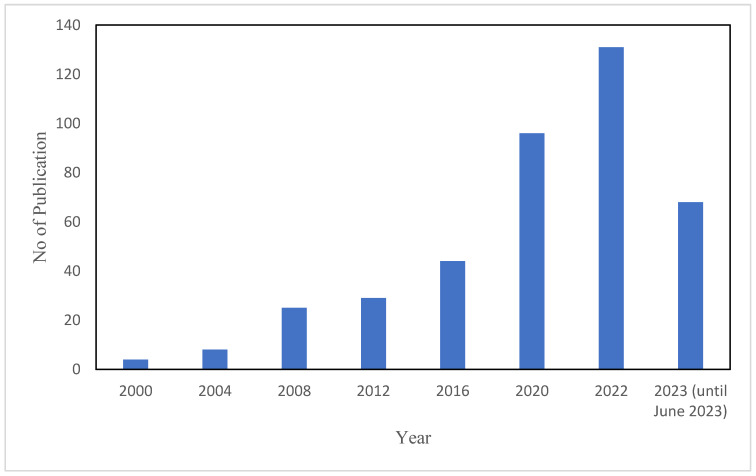
A plot of number of publications vs. year of publication was obtained from Scopus by searching for the topic ‘polymeric ionic liquid electrolyte for lithium ion batteries’ [18].

PILs research is currently in a protracted expansion period (as shown in Figure 2), with several unique properties and applications being discovered recently.

There are two main characteristics that need to be focused on to develop a good electrolyte for LIBs: good conductivity and thermomechanical properties. Numbers of PIL architectures, including, e.g., single-charged polymers, zwitterionic polymers, polyelectrolyte blends, and ion-containing block copolymers, are being investigated. Although PILs provide excellent tunable mechanical integrity, they exhibit lower ion mobility in the polymeric matrix, thus decreasing the ionic conductivity values. Researchers have concentrated on two very distinct but equally effective techniques to address the dichotomy between mechanical integrity and ion conductivities:

(i) Improve the ILs’ thermo-mechanical strength, which would allow for thinner membranes.
(ii) Improve the PILs’ ion conductivities.

Lodge et al. have pioneered the work on enhancing the mechanical integrity of IL and promised the potential of ion gels as solid electrolytes with excellent ion conductivities [19,20,21,22]. Ion gels are made of block copolymers that have been swelled with ILs, which act as the block copolymers’ solvents. Since one of the blocks in a block copolymer is still insoluble in the IL and self-assembles into aggregates, the polymer offers physical crosslinks. The synthesized soft ion gels displayed strong ion conductivities and promising elasticity (up to 350% strain and 10^3^–10^5^ Pa elastic storage modulus). Ion mobility in PILs is the topic of another effort [21].

In contrast, Drockenmuller and colleagues created 1,2,3-trizolium-based PILs with siloxane backbones to increase the ion conductivity of polymeric matrix [23]. The glass transition temperature (*T*_g_) was lowered by the siloxane backbone to as little as 100 °C, and this resulted in a promising ion conductivity even at ambient temperature (0.63 × 10^−3^ S cm^−1^). Long et al. [24] were able to obtain more than an order-of-magnitude increase in ion conductivities when switching counterions from trifluoromethane sulfonate (TfO^−^) to bis(trifluoromethane sulfonyl)imide (Tf_2_N^−^).

Imidazolium-, ammonium-, and phosphonium-based PIL materials have received a lot of attention as ion-conductive components for electrochemical devices like fuel cells, lithium batteries, actuators, and solar cells [25]. Contrary to imidazolium-based compounds, phosphonium-based ILs and PILs have not gathered much attention in the literature despite their substantial advantages compared to nitrogen-based analogues. One notable exemption in 2022 is Hofmann et al., who have studied the phosphonium-based ionic liquid electrolyte for battery application [26].

**Figure 3:**
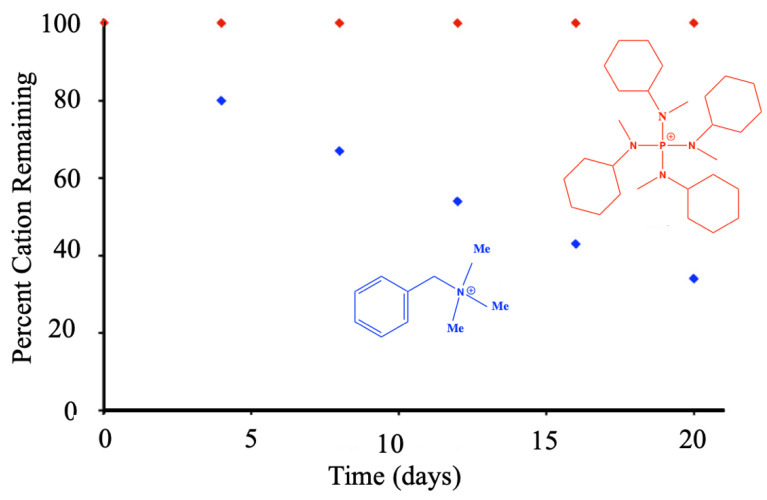
Stability of [BnNMe_3_]^+^ (blue squares) and [P(N(Me)Cy)_4_]^+^ (red squares) in 1 M NaOD/CD_3_OD at 80 °C. Reproduced from [29].

Phosphonium-based ILs and PILs exhibit enhanced thermal stability, base stability, and ion conductivities compared to nitrogen-based analogues [27,28,29]. For instance, poly(4-vinylbenzyl ammonium) and poly(4-vinylbenzyl phosphonium) homopolymers with different alkyl replacements were examined by Long et al. and it was observed that phosphonium-based PILs showed an increase in the onset of thermal deterioration of over 100 °C 45 [27]. Hoffman elimination and/or reversible Menschutkin degradation occur in ammonium salts (for benzylic protons). In contrast, phosphonium salts have higher thermal stability because they are less susceptible to both breakdown processes [27,30]. On the other hand, alkaline anion exchange membranes made of polyethylene functionalized with phosphonium pendant groups were created, according to Coates and colleagues [29]. In comparison to their ammonium counterparts, phosphonium PILs also displayed higher ion conductivities, observed at 22 × 10 ^−3^ S cm^−1^ (normalized with *T*_g_). The phosphonium PILs also showed better alkaline condition stability compared to their ammonium counterparts, as depicted in Figure 3. Thus, tetrakis(dialkylamino)phosphonium materials are promising candidates for testing in alkaline membrane fuel cell applications.

## 2. Phosphorus

Phosphorus occurs in rocks, soil, plants, and animal tissues. The several allotropes of elemental phosphorus, including the white, red, black, and purple varieties, are also well known. The alteration that best exemplifies the end result of industrial production is white phosphorus (P_4_). Phosphorus-rich molecular compounds can be created by functionalization because of P_4_’s strong reactivity and unique molecular structure. Both white and yellow are commercially available phosphorus formulations. Small amounts of red phosphorus are present in white phosphorus to form yellow phosphorus. Red phosphorus is created when white phosphorus is heated in the absence of oxygen in an inert atmosphere [31,32].

Finite resources of phosphate rock, mineralogical phosphate reserves, and irreplaceable resources cause rising research on the recycling of phosphorus. It is a crucial mineral as a nutrient for many biological processes. However, materials science also uses phosphorus compounds in many different fields. Specifically, recycling the phosphorus from the lithium-ion battery will be an important concept in the future. Nowadays, phosphorus recycling from wastewater treatment residues is a crucial strategy for protecting the world’s phosphorus supplies, and many technologies have been devised to produce pure and effective phosphorus from these wastes, which are basically produced from fertilizers. Additionally, phosphorus-bearing polymer matrices at their end-of-life accumulate in wastewater, sewage sludge, or soils. Phosphorus can be recovered after anaerobic digestion resulting in sewage sludge ashes, mainly in the form of magnesium ammonium phosphate or calcium phosphate. Sludge can be directly used as fertilizer on agricultural soils.

In recent years, the Life Cycle Assessment (LCA) has been performed to determine if recovering dissipated phosphorus by creating phosphate fertilizer based on sludge can be an effective way to prevent phosphorus depletion. Consequently, upstream sludge production was taken into account by allocating some of the environmental costs associated with wastewater treatment to sludge production. It showed that the upstream burden of sludge generation and P recovery was less environmentally benign than that of mineral phosphate fertilizers [33]. However, recovering phosphorus using struvite precipitation [34] and supercritical water oxidation [35] are alternative methods to recover phosphorus if fertilizer production is not desired.

Phosphorus plays a vital role in organic and inorganic chemical synthesis routes, including the Wittig reaction, Staudinger reaction, Arbusov and Michaelis-Arbusov reaction, Mitsunobu reaction, Horner-Wadsworth-Emmons reaction, or as multifunctional phosphine ligands in metal complex catalysts [31]. Additionally, phosphorus is widely used in the study of solid-state chemistry and materials science. It is used in steel [36], light-emitting diodes [37], and even matches. In recent years, phosphorus compounds have played an essential role in the chemical industry, e.g., as desiccants (e.g., phosphorus (V) oxide) [38], in flame retardants [39], additives [40], plasticizers [41], pesticides, or as phosphate in fertilizers [42]. One of the key properties of phosphorus compounds is their thermal resistance and flame retardancy behavior [43,44,45], which could enhance the safety issue of lithium-ion batteries. Basically, phosphorus compounds in the gas phase act as radical scavengers and solid phase char yield formation to reduce the flammability of the polymer and thus increase the material’s thermal stability.

In short, phosphonium-based PILs show great promise as novel electrolyte materials, although they have not received as much research as their ammonium analogues. Although ILs and PILs have numerous applications in gene delivery [46], antimicrobial coatings [47], gas separation [48], and conductive materials [49], there is a lack of studies on phosphonium-based PILs as electrolytes in LIB applications in the literature. This review concentrates on PIL materials to demonstrate how chemical structure affects ionic conductivity and the effect of phosphorus units on the conductivity of the end product.

## 3. Phosphonium Ionic Liquid Polymer Electrolytes in Lithium-Ion Batteries

### 3.1. Polymer Electrolytes

Polymer electrolytes are prepared from the dissolution of lithium salt with low lattice energy and bulky anions into the high molecular weight polymer host [50]. The first description of ionic conductivity in polymers with complicated alkali salts was made by Peter V. Wright and Fenton in 1973 [51]. The covalent interaction between the polymer backbones and the ionizing groups provides the basis for ionic conduction in polymer electrolytes. The polymer’s electron donor group first creates solvation with the dopant salt’s cation component before facilitating ion separation and an ionic doping mechanism. It produces ionic conductivity as a result [50].

**Figure 4:**
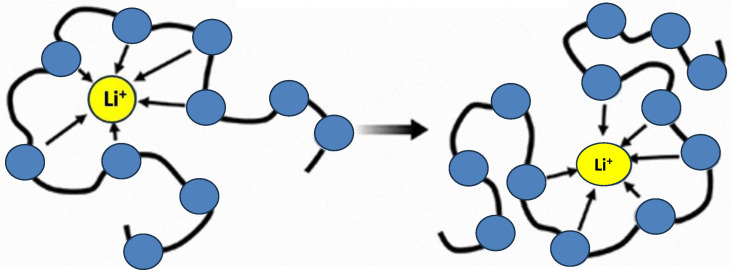
Structural representation of Li^+^ transport in PEO-doped polymer electrolytes.

The main requirement when choosing the polymer host is that the polymer must be able to pair with lithium ions as well as dissolve lithium salt to create a polymer electrolyte with high lithium-ion conductivity. The polymer’s polar groups (−O, −S, −N, −P, etc.) work well as building blocks to dissolve lithium salts. Polyethylene oxide (PEO) and its derivatives are the main subjects of most polymer electrolyte research activities. The anion and cation of the lithium salt dissociate because of the Coulombic interaction between the single electron pair of oxygens on the PEO segment and the lithium ion [52]. Lithium salt dissolves into the PEO matrix, while PEO serves as a solvent in the process. Other atoms, such as the nitrogen in the imide (−C=O)NR−(C=O)) and the sulfur in the thiol (−SH), as well as the oxygen atom (−CH_2_CH_2_O−) on the PEO chain, also play a similar role. Li^+^ cations migrate along the polymer segment, or jump from one segment to another, from one coordination point to another, when exposed to an electric field. Figure 4 illustrates the ion transport mechanism of polymer electrolytes like PEO [53].

Additionally, the flexibility of the polymer chain, the quantity of lithium ions, and the primary electric charge all affect ionic conductivity [54]. Lithium salt’s low lattice energy aids in the dissociation of a polymer with a high dielectric constant [54]. It appears necessary to use a fully amorphous polymer considerably above its glass transition temperature to ensure a large conductivity of the polymer electrolyte. Along the polymer length, the Li^+^ cations move from one coordination site to another under the influence of an electric field [55].

It is generally known that anions can reduce conductivity by generating nonconductive ion pairs [56]. The lithium-ion transference number (t_Li_^+^) is likewise lowered by the dispersion of anions [57,58]. Therefore, one of the main objectives of innovative electrolyte materials is to control anions in order to regulate these characteristics. Some specific methods for controlling anions have been studied, including electrolyte viscosity [59], high dielectric solvents [60,61] Lewis acidic electrolytes [62,63], single conducting polymers [64], and ionic liquids [65].

### 3.2. IL-Based Polymer Electrolyte

An ionic liquid is a molten salt at low temperatures, and it often consists of organic cations and inorganic anions [66]. Ionic liquids have excellent thermal stability, great electrochemical stability, and no vapor pressure due to their unique condition [65]. Although ionic liquids have excellent ionic conductivity, their low viscosity prevents them from being used directly as electrolytes. As a solid electrolyte for lithium-ion batteries, the combination of ionic liquid and polymer provides alternatives.

Higher ionic conductivity is produced when IL is added to a polymer, although this is typically accompanied by a loss of mechanical strength, especially at high temperatures. Higher mechanical strength and a smoother continuous electrolyte surface are produced by lower IL concentrations, which are better for ion transport. Therefore, the ionic conductivity and mechanical characteristics are significantly influenced by the amount of IL. Additionally, battery cycling at high temperatures typically results in the breakdown of the IL components, thus lowering performance. It increases the demand that the polymer components maintain high IL contents by one more criterion. The primary categories of IL-based polymer electrolytes include two classes: (1) ILs/polymer doped; and (2) polymeric ionic liquids (PILs) [52].

#### 3.2.1. Ionic Liquid (ILs)/Polymer Doped (Blending Technique)

Due to their comparatively strong ionic conductivity and low interfacial resistance, IL-doped polymers have received much research as semi-solid electrolytes [67,68]. For example, a gel polymer electrolyte containing phosphonium ionic liquid has been prepared [69]. A mixture of lithium salt and trihexyl (tetradecyl)phosphonium bis(trifluoro methane) sulfonamide IL was doped into a polymer host network formed of an epoxy prepolymer and an amine hardener. The authors showed that the inclusion of electrolyte has a significant impact not only on the final properties of epoxy networks but also on the kinetics of polymerization, particularly the thermal, thermo-mechanical, transport, and electrochemical properties. Thus, polymer electrolytes with exceptional thermo-mechanical and thermal stability (>300 °C) have been created. At 100 °C, an ionic conductivity of 0.13 × 10^−3^ S cm^−1^ was attained.

**Figure 5:**
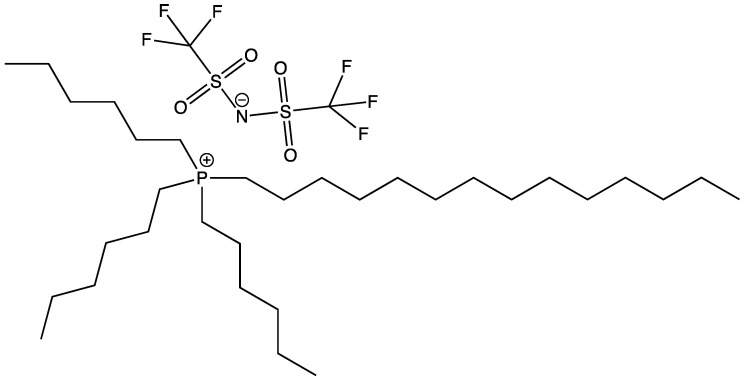
The structure of tri-hexyl tetradecyl phosphonium bis(trifluoromethyl sulfonyl)imide ionic liquid.

Singh et al. have prepared polymer electrolytes using poly(ethylene oxide) (PEO), lithium bis (trifluoromethyl sulfonyl) imide (LiTFSI), and ionic liquid (IL) trihexyl tetradecyl phosphonium bis(trifluoromethyl sulfonyl)imide (depicted in Figure 5) with different concentrations of IL (0 wt%, 10 wt%, 20 wt%, 30 wt%, and 40 wt%). They discovered the differences in crystallinity, melting temperature (*T*_m_), glass transition temperature (*T*_g_), thermal stability, and ionic transport behavior of the prepared polymer electrolyte when the LiTFSI salt and different concentrations of IL were incorporated in the pristine polymer PEO. It has been discovered that the ionic conductivity of the solid polymer electrolyte increases when the trihexyl tetradecyl phosphonium bis(trifluoromethyl sulfonyl) imide IL content increases in polymer electrolytes up to 20 wt% IL; the optimum conductivity observed was 4.2 × 10^−5^ S cm^−1^ and the Xc (degree of crystallinity) also decreases up to 20 wt% IL [70].

**Figure 6:**
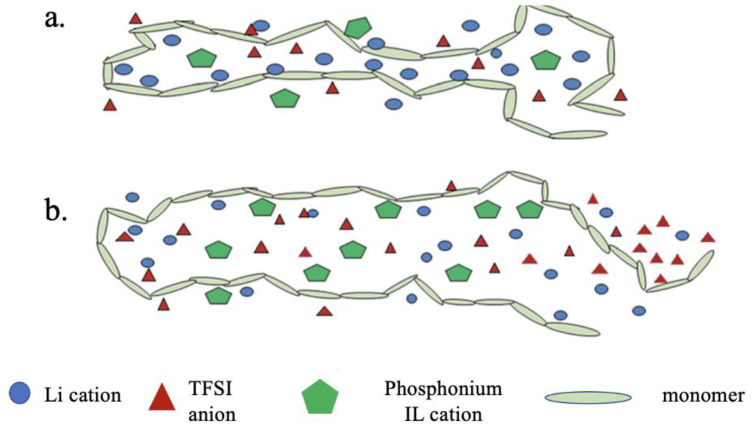
Schematic representation of the flexibility of polymer chains: PEO + 20 wt% LiTFSI + X% IL (**a**) X = 10%, (**b**) X = 20%. Reproduced from [70].

In Figure 6, a phenomenological model is shown to explain how the amount of IL causes a change in the flexibility of the polymer chain, which in turn affects ion mobility. The plasticizing activity of the IL causes the polymer chain to become more flexible and unfold at lower IL levels (10 wt% of IL) in polymer electrolyte films, as seen in Figure 6. (a). The polymer chain gets more flexible after 20 wt% incorporation of IL (Figure 6b).

**Figure 7:**
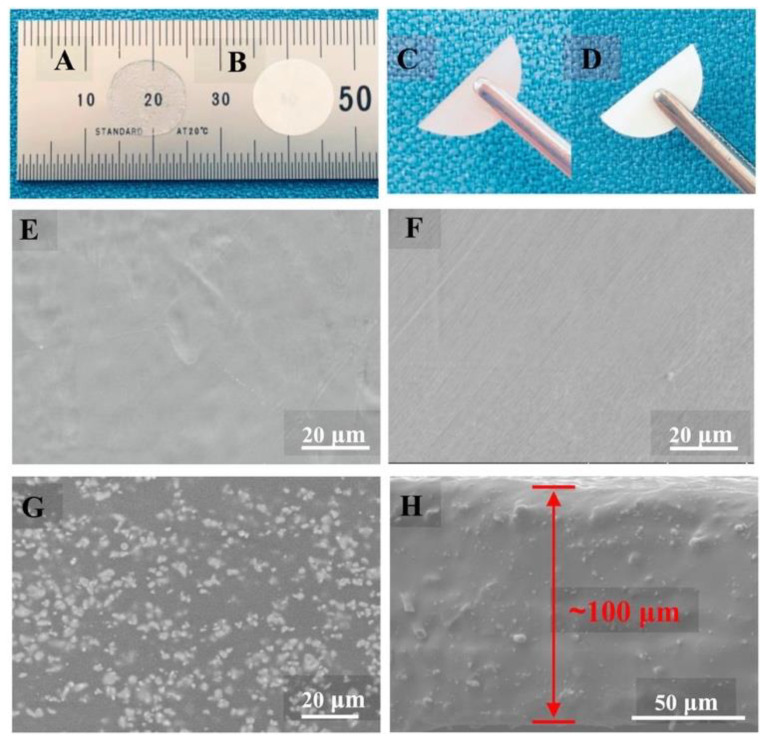
Photographs of the prepared (**A**,**C**) PEO_8_-LiTFSI-15% TBPHP and (**B**,**D**) PEO_8_-LiTFSI-TBPHP-12.5% LLZTO SPE membranes at flat and bended states; The typical surface morphologies of (**E**) PEO_8_-LiTFSI, (**F**) PEO_8_-LiTFSI-15% TBPHP, and (**G**) PEO_8_-LiTFSI-TBPHP-12.5% LLZTO; (**H**) cross-sectional SEM image of PEO_8_-LiTFSI-TBPHP-12.5% LLZTO. Reproduce from [76] with permission. Copyright 2019 Elsevier.

To improve the conductivity, mechanical, and electrochemical properties of polymer electrolytes, hybrid-based polymer electrolytes have been developed [71,72,73,74,75]. Hybrid electrolytes are electrolytes with more than two components. Developing a solid-state electrolyte PEO_8_-LiTFSI-TBPHP (tetrabutyl phosphonium 2-hydroxypyridine)-12.5% and 15% LLZTO (Li_6.4_La_3_Zr_1.4_Ta_0.6_O_12_) was recently reported by Xie et al. using a solvent-free method (Figure 7) [76].

Ionic liquids and ion-conducting ceramics include TBPHP and LLZTO. The IL’s cation and anion may efficiently raise the lithium-ion transference number (t_Li_^+^ = 0.63) and decrease the PEO crystallinity. The addition of LLZTO can simultaneously increase the thermal and electrochemical stability (up to 5 V vs. Li^+^/Li), conductivity (2.51 × 10^−4^ S cm^−1^ at 30 °C and 9.39 × 10^−4^ S cm^−1^ at 50 °C), and mechanical strength, respectively [76].

**Figure 8:**
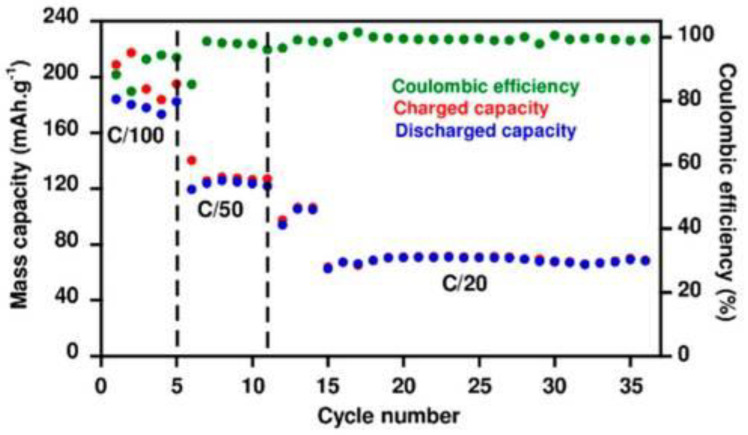
Cycling performance of the Li|PPO [TFSI]−70|LiFePO_4_ cell at indicating rates at 100 °C. Reproduced from Ref. [69]. Copyright MDPI.

Several findings point to a promising gelled electrolyte-based phosphonium-based IL for lithium-ion batteries in the future. New gelled electrolytes were prepared by Melody and coworkers [69] that consist of a mixture containing phosphonium ionic liquid (IL) composed of trihexyl(tetradecyl)phosphonium cation combined with bis(trifluoromethane)sulfonimide counter anions (PPO[TFSI]) and lithium salt, confined in a host network made from an epoxy prepolymer and amine hardener. It was shown that the addition of electrolyte affects not only the final properties of epoxy networks, particularly their thermal, thermomechanical, transport, and electrochemical properties, but also the kinetics of polymerization. As a result, polymer electrolytes with excellent thermomechanical and thermal stability (>300 °C) have been created. At 100 °C, an ionic conductivity of 0.13 × 10^−3^ S cm ^−1^ was also attained. Its electrochemical stability was 3.95 V vs. Li^0^/Li^+^, and even after 30 cycles, the assembled cell made of Li|LiFePO_4_ exhibited stable cycle properties as shown in Figure 8.

**Figure 9:**
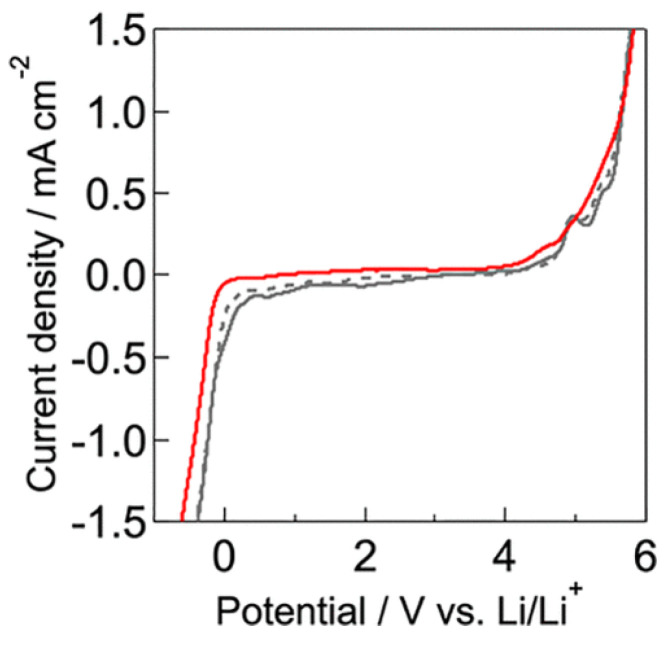
Electrochemical windows of 3 wt% TetraPEG ion gel (red) and the corresponding IL solution with 1-ethylimidazole, C_2_Im (gray solid) and without C_2_Im (gray dotted) at 298 K; scan rate: 5.0 mV s^−1^. Reproduced with permission from Ref. [77].

Kenta and coworkers [77] have synthesized an ion gel electrolyte using a TetraPEG network in an ionic liquid (IL) solution based on phosphonium by using the “salting-in” effect of a Li salt. PEG chains are insoluble in phosphonium-based ILs (such as triethylpentylphosphonium bis(trifluoromethanesulfonyl)amide, [P_2225_][TFSA]), but TetraPEG was completely soluble in [P_2225_][TFSA] after the addition of LiTFSA. The optimal concentrations for making polymer gel electrolytes were determined by examining the Li salt and polymer concentration dependences for PEG solubility in a [P_2225_][TFSA] system. The final TetraPEG gel electrolyte polymer network demonstrated high ionic conductivity (2.78 × 10^−1^ S cm^−1^ at ambient), comparable to the corresponding solution electrolyte. It was almost defect-free. A wide electrochemical window around 4.2 V vs. Li^0^/Li^+^ (depicted in Figure 9) and the presence of LiTFSA/[P_2225_][TFSA] in the TetraPEG gel electrolyte allowed for the use of conventional metal negative electrodes in the reversible Li deposition/dissolution reaction.

**Figure 10:**
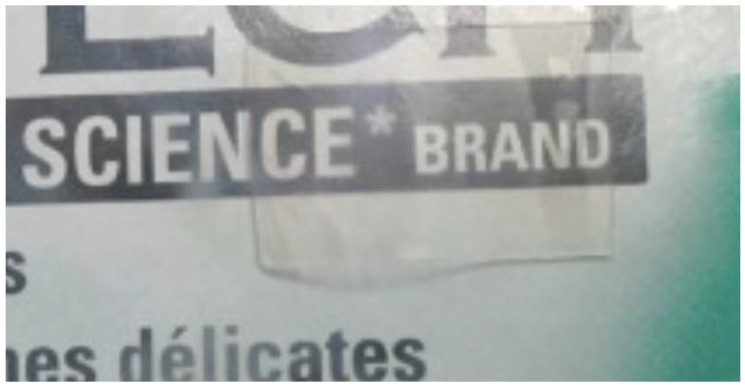
Physical state of the PDADMA-FSI mixture at 60:40 wt%. Reproduced with permission from Ref. [78].

Poly(dialyldimethylammonium) (PDADMA) is the common polymer host used in ion gel or composite polymer electrolytes for lithium batteries. Forsyth et al. [78] have synthesized the novel ternary systems by combining trimethylisobutylphosphonium bis(flurosulfonyl)imide ([P111i4FSI]), PDADMA FSI, and lithium salt at high concentrations. A free-standing flexible and transparent membrane (Figure 10) with a conductivity of 0.49 × 10^−3^ S cm^−1^ at 40 °C and a Li^+^ transference number of 0.21 at 50 °C is produced using an electrolyte (3.8 m LiFSI in P111i4FSI (LiFSI/IL)): PDADMA FSI mixture at a 60:40 wt%.

**Figure 11:**
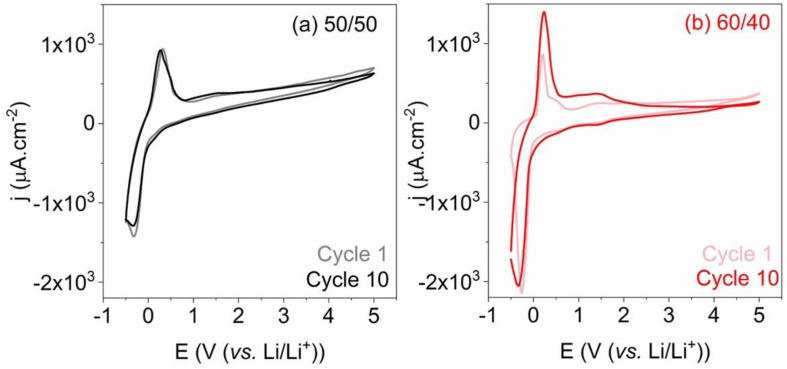
(**a**) Linear sweep voltammograms (1st and 10th cycles) for two ternary systems, 50:50 wt% (FSI system) and (**b**) 60:40 wt% (FSI system), at a stainless steel (SS) working electrode with a potential sweep rate of 20 mV∙s^−1^ at 50 °C. Reproduced with permission from Ref. [78].

Cyclic voltammetry (CV) was used to evaluate the electrochemical stability of the flexible films. The linear sweep voltammograms for the FSI-based ternary systems, 50:50 wt% and 60:40 wt%, are shown in Figure 11. The information supports the reasonable maintenance of Li^+^ reduction and oxidation over ten cycles. When comparing the 60:40 wt% (FSI system) to the 50:50 wt% (FSI system) in the first cycle, the current density at the reduction peak is higher, while the current density at the oxidation peak is similar for both compositions. It’s interesting to note that after 10 cycles, the current density at the oxidation peak for the 60:40 wt% (FSI system) significantly increases, rising from roughly 0.8 to 1.4 mA.cm^2^, indicating an increase in coulombic efficiency [78]. The various types of IL/polymer doping have been tabulated in Table 1.

#### 3.2.2. Polymeric Ionic Liquids (PILs)

It is possible to create polymeric ionic liquids (PILs) by either directly polymerizing an IL-based monomer or by polymerizing a modified polymer with an IL monomer. PIL membranes have attracted a lot of interest recently by fully utilizing the unique properties of ionic liquids and polymers.

Generally, there are two types of PILs: the anion-backboned polymer and the cation-backboned polymer. If the polymer backbone is formed of anions, the ionic conductivity is provided by the mobility of cations, and vice versa. Since both the anion and the cations of ILs may be easily selected, this presents a special possibility for developing selective ionic conductivity by a particular ion [85]. While the flexibility of π-conjugated electrons, which promotes electrical conductivity, is the primary characteristic of electronically conductive polymers, the extensive presence of ions throughout the PIL structure ensures ionic conductivity.

PILs, like conductive polymers, can serve as an effective electroactive material matrix in a variety of electrochemical systems [86]. In contrast, if PILs are widely used as a matrix for the fabrication of nanocomposites, the second component can also contribute to the ionic conductivity of PILs. PIL nanocomposites have an ionic conductivity of up to 0.1 × 10^−3^ S cm^−1^ [87]. We will focus on phosphonium-based polymeric ionic liquids in the following section.

## 4. Phosphonium-Based Polymeric Ionic Liquid Electrolyte

Some of the important factors have to be to taken into account when selecting a polymer host for PEs include better coordination with anions, thermal stability, and high oxidation states [88]. These factors are all present in phosphorus-based polar functional groups connected with ILs and form functional polymers. Thus, a new family of phosphonium-based materials with unique properties and intriguing applications has recently emerged because of the introduction of the functional groups associated with ILs into functional polymers.

### 4.1. Synthesis Route and Conductivities

**Figure 12:**
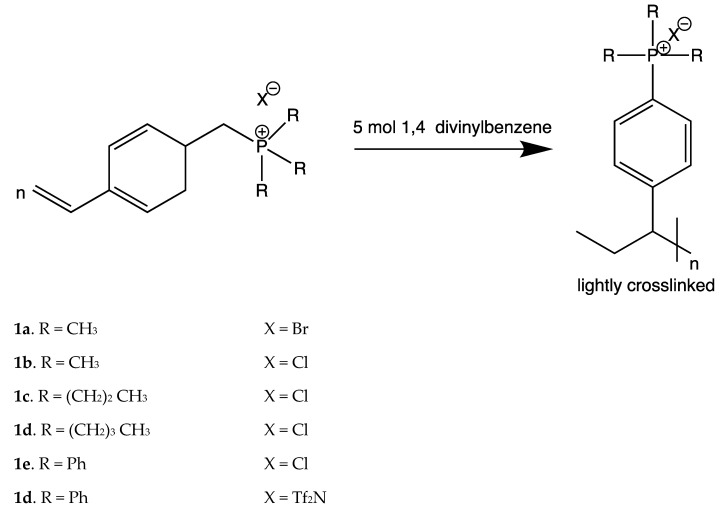
Chemical compositions of the related polymers and the functional monomers used in phosphonium-based polyvinyl benzene. Reproduced from [89].

Typically, there are two alternative approaches to making polymeric ionic liquids with phosphonium cations: Using functional monomers [89] or through post-modification functionalization (grafting) [90]. The standard method for creating cationic phosphonium monomers involves a straightforward nucleophilic substitution between a haloalkane and a tertiary phosphine [89]. Gabriel and co-workers successfully synthesized trimethyl-(4-vinyl-benzyl)-phosphonium bromide (**1a**), tripropyl-(4-vinyl-benzyl)-phosphonium chloride (**1b**), triphenyl-(4-vinyl-benzyl)-phosphonium chloride (**1c**), tributyl-(4-vinyl-benzyl)-phosphonium chloride (**1d**), triphenyl-(4-vinyl-benzyl)-phosphonium chloride (**1e**), triphenyl-(4-vinyl-benzyl)-phosphonium bis(trifluoromethyl sulfonyl)amide (**1f**) (as shown in Figure 12) via a functional monomer route. Initial solutions were prepared with 10 wt% monomer in methanol, 5 wt% cross-linker 1,4- divinylbenzene, and 1 wt% 2-hydroxy-2-methylpropiophenone (HMP) photoinitiator. A germanium crystal was pipetted with the necessary amount of monomer solution. A thin coating of monomer, a cross-linker, and a photo initiator was left when the solvent evaporated at ambient temperature and atmospheric pressure. The pre-polymerized film’s FT-IR spectrum was then recorded. After three argon purges and photopolymerization with 365 nm light lasting 30 min, the coated Ge crystal was subsequently enclosed inside the polymerization chamber. The degree of polymerization was then determined by taking an IR spectrum of the polymerized film and comparing it to the pre-polymerized film.

Monomer stability tends to be a limiting factor in the suitability of a functional monomer route. Post-polymerization functionalization avoids potential monomer stability issues or polymerization hurdles, but it remains disadvantageous to achieve 100% functionalization due to steric and neighboring group effects [73].

**Figure 13:**
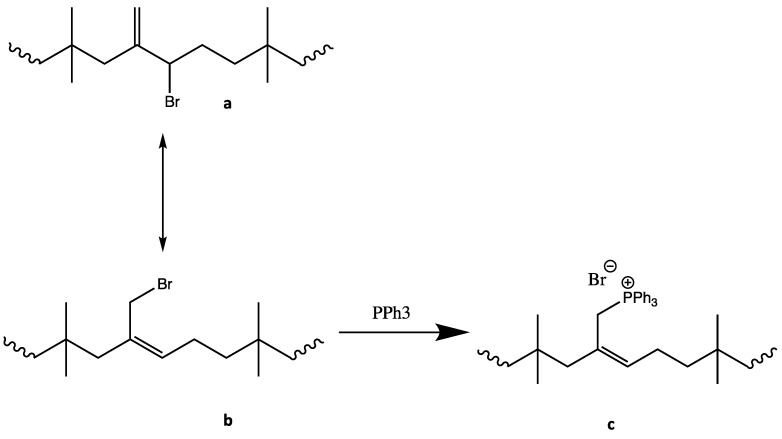
Chemical reaction of phosphonium polymeric ionic liquid via postmodification, (**a**) exo-methylene allylic bromide, (**b**) (E,Z)-endo isomer and (**c**) phosphonium bromide (IIR-PPh_3_Br. Reproduced from reference [90] with permission.

Through a post-modification functionalization route, Parent et al. [90] have synthesized a class of polymeric ionic liquids by displacing bromide from brominated poly(isobutylene-*co*-isoprene) (BIIR) with triaryl phosphine nucleophiles. Figure 13 shows the chemical reaction of phosphonium-based polymeric ionic liquid.

**Figure 14:**
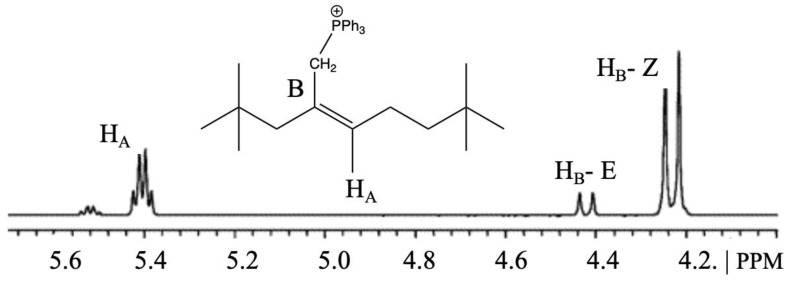
Downfield region of ^1^H NMR spectrum of model compounds (CDCl_3_). Reproduced from reference [90].

Exo-methylene allylic bromide (a), a kinetically preferred bromination product in BIIR, can change into the more stable (E,Z)-endo (b) isomer at high temperatures (Figure 9) [90]. Thus, the authors proved by means of ^1^H NMR (Figure 14) spectroscopy that only a (E,Z)-endo allylic phosphonium bromide is formed; neither the model nor the BIIR substitution products show any signs of an exo-methylene isomer similar to exo-methylene allylic bromide (a).

**Figure 15:**
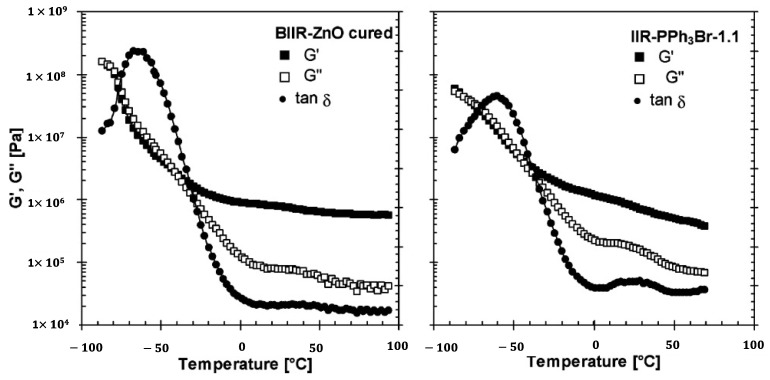
Storage, loss, and phase angle data for ZnO-cured BIIR, IIR-PPh_3_Br, as a function of temperature (1 Hz, 1° strain). Reproduced from reference [90].

The resultant phosphonium bromide (IIR-PPh_3_Br) PILs have dynamic mechanical properties like thermoset vulcanizates, but the elastomeric network is the consequence of ion-pair aggregation. When PPh_3_ is quaternized by interaction with BIIR, elastomeric ionomers are created that have mechanical qualities similar to those of ZnO-cured vulcanizates. Figure 15 displays the dynamic mechanical characteristics of IIR-PPh_3_Br as a function of temperature. For comparing the performance of the ionomers and a non-ionic, cross-linked polymer network, a ZnO-cured sample of BIIR is used as a control material. The data only slightly differ between the *T*_g_s of BIIR-ZnO-cured and PPh_3_Br-IIR.

Basically, the synthesis route that gives more hermos-mechanical strength and conductivity, whether it is functionalized monomer or post-modification, depends on several factors, including the type of functional group(s) being introduced, the polymer structure, and the processing conditions. In general, functionalized monomers can lead to higher thermo-mechanical strength because they allow for a more homogeneous distribution of the functional group(s) throughout the polymer chain. This can result in a more ordered and interconnected network of cross-links and conductive pathways, which can lead to higher mechanical strength and conductivity, respectively. However, post-modification can also lead to high thermomechanical strength and conductivity if the functional groups are introduced in a way that allows for a high degree of cross-linking or interconnectivity between the polymer chains. This can result in a network that is similar to that obtained with functionalized monomers. Ultimately, the choice between functionalized monomers and post-modification will depend on the specific requirements of the application, the availability of the starting materials, and the ease of the synthetic route. Both approaches have the potential to lead to high thermomechanical strength, and the choice should be made based on careful consideration of these factors.

In contrast, polymers analogous to ionic liquids show greater mechanical strength, and thus increasing the mechanical properties of the electrolytes can prevent electrical shorts and uncontrolled dendritic lithium penetration [17]. A well-controlled synthesis process of PILs can produce materials with a dense and ordered well-defined architecture at the nanoscale, resulting in improved conductivity [91]. A well-defined microstructure can enhance conductivity by controlling charge carrier units and salt types. Precise control of impurity levels and defects such as voids and dislocations can result in materials with improved strength and conductivity. Polymer segmental motions possessing mobility of ions in the well-defined matrix improve the ionic conductivity [92].

It has to be mentioned that in PILs, “free” counterions dominantly drive ion conductivity, with confined ions typically contributing very little to this property. Similar to viscosity in ILs, glass transition temperatures (*T*_g_) in PILs typically have an inverse connection with ion conductivities [30,69,92]. Higher ion conductivities are a result of lower *T*_g_, which corresponds to more flexible matrices for counterions. There are two main methods that help lower *T*_g_. Bulkier ions introduce more free volume to decrease *T*_g_ from the perspective of free volume. Ions serve as pendant groups in this situation, and studies have shown that larger pendant groups typically cause a decrease in *T*_g_ by introducing more free volume [93,94,95].

On the other hand, electrostatic interactions are another factor to consider because they operate as physical cross-linking sites in polymer matrices to prevent mass transfer. Therefore, weak physical crosslinks and low *T*_g_ result from weak electrostatic interactions [93]. Nevertheless, higher conductivities are not exclusively dependent on low *T*_g_. Small structural changes with little change in *T*_g_ can reduce electrostatic interactions and increase ion conductivity. Regarding this, phosphonium-based PILs offered tremendous promise for increasing ion conductivity in comparison to their nitrogen-based analogs without sacrificing mechanical qualities (decreased *T*_g_) [27].

Additionally, the crystallinity of the polymer may affect its conductivity value. The crystalline region refers to the extent to which a polymer has ordered. Polymers possessing crystalline domains with tightly packed chains can act as physical barriers, and these regions typically have lower mobility for both ions and polymer segments. As a result, the overall ionic conductivity of the polymer electrolyte may be reduced. The amorphous regions of the polymer, on the other hand, tend to have higher mobility for ions and enable faster ionic conduction. Processing techniques such as annealing, plasticizers, additives, or copolymers with high amorphous content can alter the crystalline structure of the overall matrix. This can affect the crystallinity and alignment of polymer chains, leading to increased ion mobility and higher conductivity. Previously, it was also shown that *T*_g_ has a strong relation with ionic conductivity [96]. Segmental motion of the chains results in increasing ion conduction through the flexible backbone-backbone distance.

Various studies regarding the conductivity values of phosphonium PILs were summarized in Table 2. From the polymer backbone, we can observe that the PILs consisting of flexible backbones exhibit higher ionic conductivity values.

### 4.2. Analytical Tools

Once the phosphonium polymer electrolyte is synthesized, it is important to characterize its properties to assess its suitability for the intended application. Here are some key characterizations of phosphonium-based polymer electrolytes after the synthesis:

Ionic conductivity measurement: This is a crucial characterization technique to evaluate the efficiency of the electrolyte in transporting ions. Conductivity measurements can be performed using techniques such as impedance spectroscopy or electrochemical impedance spectroscopy (EIS) [100]. Depolarized dynamic light scattering broadband dielectric spectroscopy can also be used to study the mechanisms of ionic transport and segmental dynamics in these materials [101]. Walden plot analysis can be conducted to analyze the classification of ionic conductors by analyzing the molar conductivity of a given electrolyte with viscosity [102].

Thermal stability analysis: Differential scanning calorimetry (DSC) [103] and thermogravimetric analysis (TGA) [104] can be used to study the thermal stability of the polymer electrolyte. These techniques help determine the onset of thermal degradation and the temperature range over which the electrolyte remains stable.

Structural analysis: IR and Raman spectroscopies can provide information about the molecular structure and dynamics of PILs [105]. Nuclear magnetic resonance spectroscopy (NMR) can also provide information about the connectivity of polymer chains, the arrangement of ionic groups, and the mobility of polymer segments [106,107].

X-ray Diffraction (XRD) is employed to analyze the crystalline structure and phase behavior of PILs [108]. It can determine the degree of crystallinity, crystal structure, and phase transitions, providing insights into the solid-state properties of the material. Small-Angle X-ray Scattering (SAXS) and Small-Angle Neutron Scattering (SANS): These techniques are used to investigate the structural arrangement and morphology of PILs on the nanoscale [109]. They provide information about the size, shape, and ordering of microdomains or aggregates within the material.

Mechanical properties: Mechanical tests, including tensile strength, elongation at break, and Young’s modulus, can assess the mechanical stability and flexibility of the polymer electrolyte [108,110]. PILs with higher elastic moduli are generally desired as they provide better mechanical strength and help prevent electrode-electrolyte separation or fracture during battery operation. High tensile strength is desirable to ensure the mechanical integrity of the electrolyte under the mechanical stresses experienced during battery assembly, cycling, and thermal variations. Flexibility and ductility are important for maintaining good contact between the electrolyte and electrode materials, especially in flexible or stretchable battery designs. In lithium-ion batteries, good adhesion between the polymer electrolyte and electrode materials is crucial to ensure efficient ion transport and prevent delamination or detachment of the electrolyte from the electrode interfaces. PILs with strong adhesion properties can enhance the mechanical stability and overall performance of the battery [97].

### 4.3. Comparison between Phosphonium-Based PILs and Nitrogen-Based PILs

There have been very few studies on phosphonium-based polyelectrolytes compared to ammonium-based polyelectrolytes up to this point [98]. In contrast, PILs based on phosphonium were discovered to have greater thermal stability and ionic conductivity than their ammonium-based counterparts. Long et al. have synthesized high-molecular-weight polymerized ionic liquids using conventional free radical polymerization and anion metathesis of ammonium and phosphonium styrene (PILs) [111]. The thermal stabilities of phosphonium-based polyelectrolytes containing Tf_2_N^−^ counterions are significantly higher (437 °C) than those of ammonium analogs (330 °C). Additionally, ionic conductivities of PILs containing phosphonium PILs show larger values than ammonium analogs when measured using impedance spectroscopy (summarized in Table 3).

**Scheme 1:**
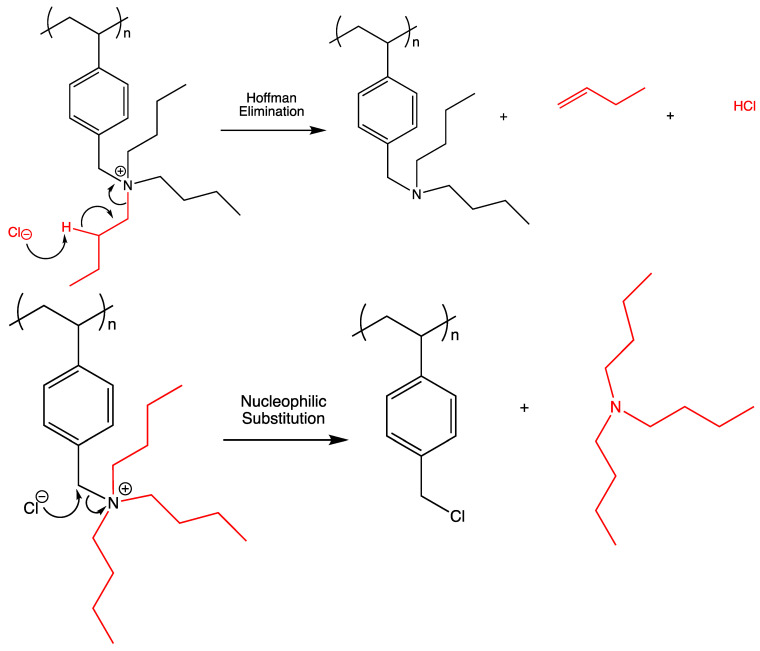
Thermal degradation of ammonium polyelectrolytes through either a Hoffman elimination or a reverse nucleophilic substitution mechanism. Reproduced from reference [27].

Since nitrogen-based PILs undergo thermal degradation through either Hoffman elimination or reverse nucleophilic substitution (Scheme 1), phosphonium-based PILs demonstrated greater thermal stability than their nitrogen-based counterparts.

**Scheme 2:**
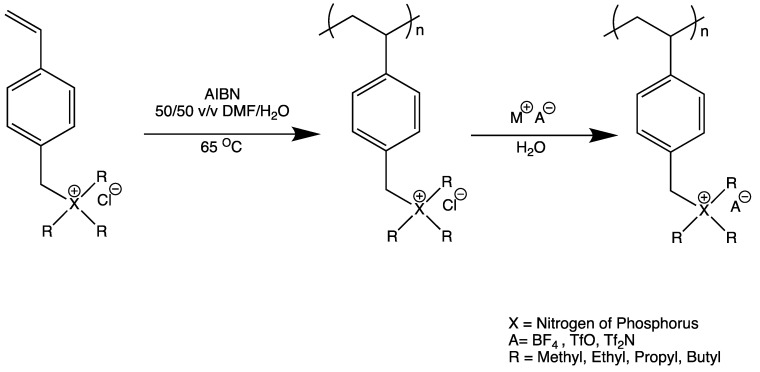
PILs containing ammonium- and phosphonium-based polymers. Reproduced from reference [27].

The comparison of phosphonium-based PILs and nitrogen-based PILs has been done by Long et al. [27]. Ammonium and phosphonium-based PILs were produced via traditional free radical polymerization of styrenes with varying alkyl substituent lengths (Scheme 2). The PILs included: poly[trimethyl-(4-vinylbenzyl) phosphonium chloride] (PTMP-Cl), poly[triethyl-(4-vinylbenzyl)phosphonium chloride] (PTEP-Cl), poly[tripropyl- (4-vinylbenzyl)phosphonium chloride] (PTPP-Cl), poly[tributyl-(4-vinylbenzyl)phosphonium chloride] (PTBP-Cl), poly[trimethyl-(4-vinylbenzyl)ammonium chloride] (PTMA-Cl), poly[triethyl-(4-vinylbenzyl)ammonium chloride] (PTEA-Cl), poly[tripropyl-(4-vinylbenzyl)ammonium chloride] (PTPA-Cl), and poly[tributyl-(4-vinylbenzyl)ammonium chloride] (PTBA-Cl).

**Figure 16:**
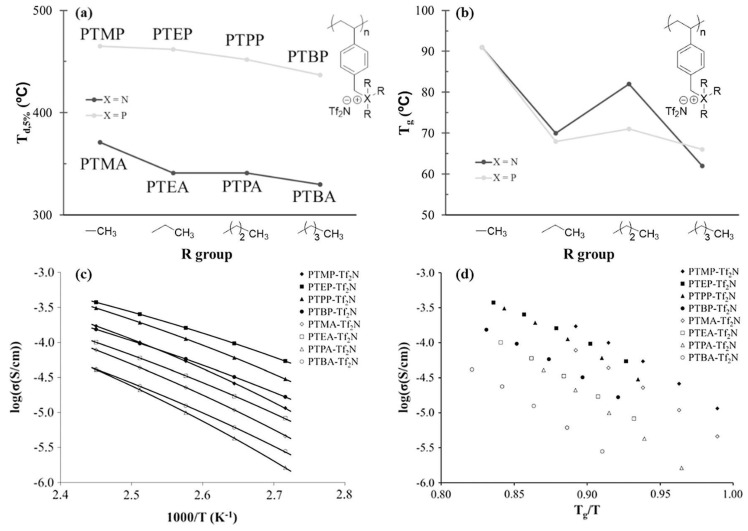
Comparison of (**a**) thermal stability, (**b**) *T*_g_, (**c**) temperature-dependent ion conductivities, and (**d**) ion conductivities normalized with *T*_g_ of P-based PILs and N-based PILs. Reproduced from reference [14].

As shown in Figure 16a, significantly higher thermal breakdown temperatures have been found for phosphonium-based PILs after synthesizing two series of styrene PILs with either quaternary ammonium or phosphonium cations (94% yield). Phosphonium polyelectrolytes containing Cl^−^ counterions display significantly higher thermal stabilities (>370 °C) compared with ammonium analogues (<220 °C). Anion exchange to BF_4_^−^, TfO^−^, and Tf_2_N^−^ shows the improvement in terms of the thermal stability of all the PILs. When compared to their nitrogen-based analogues, phosphonium-based PILs showed similar *T*_g_ (Figure 16b) but significantly greater ion conductivities (Figure 16c), suggesting the potential for P-based PILs to serve as perfect electrolytes or ion exchange membranes with strong mechanical integrity. Although there is not a sufficiently significant variation in size between nitrogen and phosphorus to noticeably alter the *T*_g_, P-based PILs have weaker electrostatic interactions than N-based PILs, which leads to higher ion conductivities [14].

Basically, nitrogen has a partial negative charge in ammonium cations because its electronegativity is higher than that of the α-carbons. The electrostatic interaction mostly takes place between counter anions and positively charged α–carbons. In contrast, in phosphonium cations, phosphorus has a partially positive charge as its electronegativity is lower than carbons, and eventually it produces slightly negatively charged α-carbons. These negatively charged α-carbons effectively shield the positive charge on phosphorus and reduce the electrostatic interactions; thus, phosphonium-based PILs make the single ions diffuse more freely, resulting in higher ionic conductivity for phosphonium-based PILs. Colby et al. have calculated the charge distribution of three types of cations: Bu_4_P^+^, Bu_4_N^+^, and BuMeIm^+^. The data have been summarized in Table 4 [93].

On the other hand, the following could be another reason why phosphonium-based PILs have better ionic conductivities than their ammonium analogues: The ammonium-based PILs described here may exhibit associative diffusion, in which the ion pair likely diffuses together as a noncharged species and does not participate in direct current ionic conduction. This is because the hydrogen bond interactions in these materials are stronger [112]. While the equivalent phosphonium-based PILs’ weaker hydrogen bond interactions allow the individual ions to circulate more freely, leading to better ionic conductivity for phosphonium-based PILs [113].

**Scheme 3:**
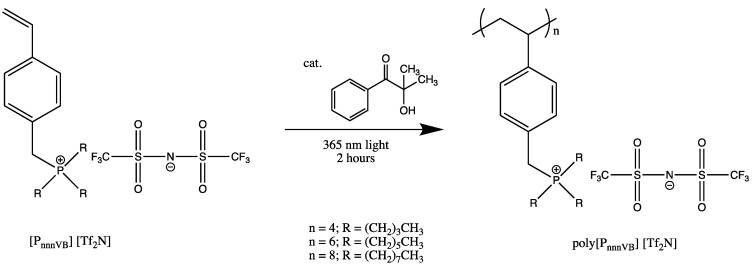
Radical photopolymerization of neat [P_nnnVB_][Tf_2_N] monomers to form the poly([P_nnnVB_][Tf_2_N]) PILs and free-standing membranes tested in this work. Reproduced from reference [25].

Substituents might affect the ion conductivity as well. *T*_g_ changes significantly as alkyl substituent length varies. As depicted in Figure 16b, when methyl R groups (PTMP) were replaced with ethyl R groups (PTEP), *T*_g_ dropped from 91 °C to 68 °C. The temperature at which the alkyl substituents continue to elongate in P-based PILs changes very little (68 °C, 71 °C, and 66 °C for ethyl, propyl, and butyl, respectively). The shorter alkyl substituents displayed higher ion conductivities at the same temperature except for methyl substituted polymers, probably because their *T*_g_ was higher than that of the other PILs by 20 °C (Figure 16c). After adjusting for *T*_g_, the ion conductivities consistently followed the predicted pattern: methyl > ethyl > propyl > butyl (Figure 16d). This tendency explains higher mobility and ion content from smaller size and reduced non-ionic content, respectively, and is consistent with ILs. Gin, Noble, and colleagues investigated the identical phosphonium-based PILs with longer alkyl substituents (hexyl and octyl) (Scheme 3), and they noticed a steady decrease in *T*_g_ [25].

**Figure 17:**
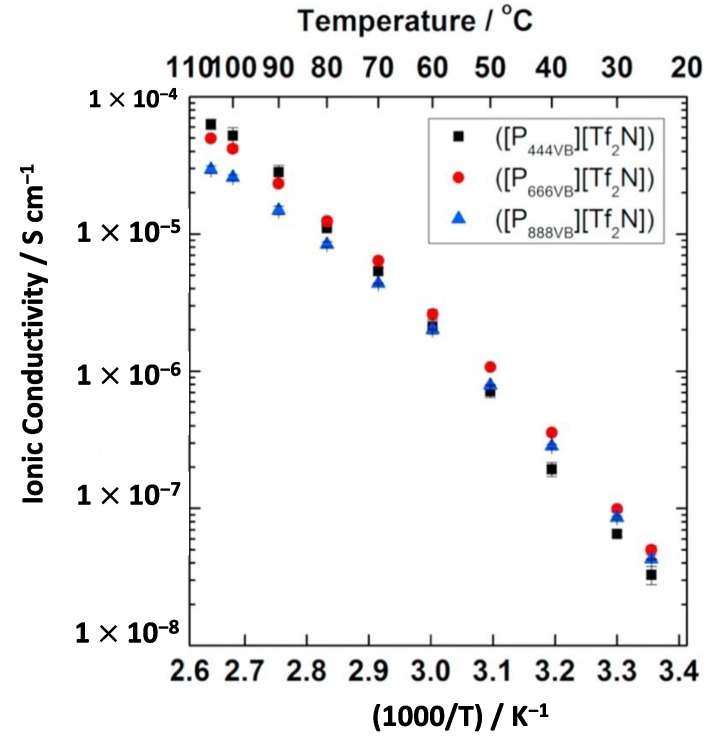
Behaviour of neat poly([P_444VB_][Tf_2_N]), poly([P_666VB_][Tf_2_N]), and poly([P_888VB_][Tf_2_N]) films between 25 and 105 °C. Reproduced from reference [25].

A temperature range of 25 to 105 °C was used to assess the ionic conductivities of the poly([P_444VB_][Tf_2_N], poly([_P666VB_][Tf_2_N], and poly([P_888VB_][Tf_2_N]) membranes. Regardless of the length of the phosphonium group alkyl chain, the ionic conductivity was quite similar for all the membranes and generally rose from 10^−8^ to 10^−4^ S cm^−1^ as the temperature increased from 25 to 105 °C (Figure 17). They found that, at 25 °C, the butyl substituent group in poly([P_444VB_][Tf_2_N]), the shorter alkyl chain length allows for a higher cation-anion attractive contact, which restricts ion mobility at lower temperatures, shows lower ionic liquid compared to the longer substituent due to the shorter alkyl chain length allows for a higher cation-anion attractive contact, which restricts ion mobility at lower temperatures. As the temperature rises, the strength of the cation-anion interaction becomes less significant, and the size of the cationic species bound to the polymer becomes more of a limiting factor for ion mobility (i.e., as a function of increasing cation alkyl chain length), which is consistent with the correlation seen at temperatures below 90 °C [25].

In short, there is a significant difference in the cation structure depending on whether the cationic atom is nitrogen or phosphorus due to differences in atomic radius and electronegativity. Over other IL types, phosphonium ILs unquestionably offer, in some circumstances, several benefits, including greater thermal stability, lower viscosity, and greater stability in strongly basic or strongly reducing conditions. Phosphonium polyelectrolytes showed improved thermal stability because of their resistance to Hofmann elimination.

### 4.4. Counter-Ion Effect in Conductivity

**Figure 18:**
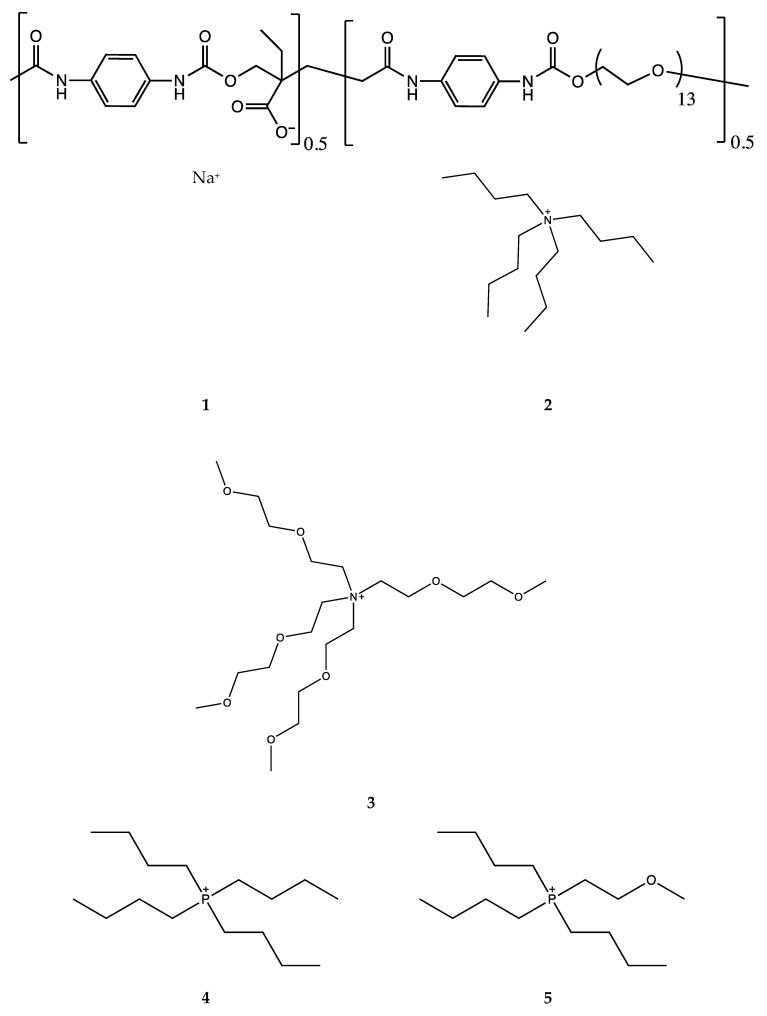
Chemical structures of polyurethane anionomers and cations studied: (1) Na^+^, (2) Bu_4_N^+^, (3) (MOEOE)3MeN^+^, (4) Bu_4_P^+^, (5) Bu_3_(MOE). Reproduced from [93].

Enhancing PILs’ ion conductivity effectively involves counterion exchange. This is accomplished through controlling the electrostatic interactions as well as plasticization. Bulky counterions produce lower *T*_g_s and more free volume, which allow mass transfer (ion conductivity). As a result of weaker electrostatic interactions, counterions with a higher delocalized charge density exhibit high ion conductivities [93]. Colby et al. synthesized the polyurethane-carboxylate ionomers with various counter cations (structures illustrated in Figure 18). The effect of counter cations on *T*_g_ and ion conductivity has been investigated [93].

**Figure 19:**
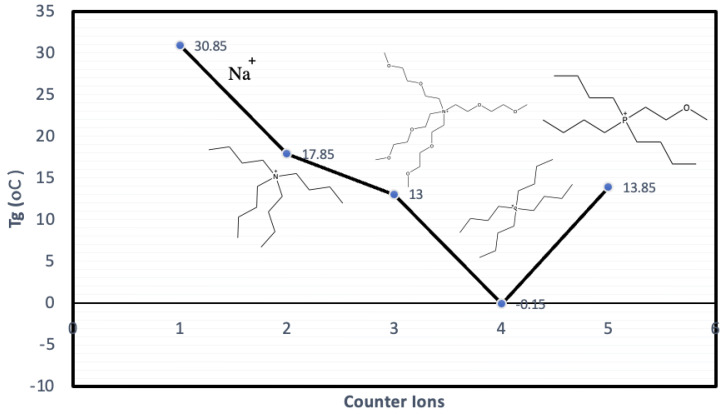
The influence of different counter cations on *T*_g_. (1) Na^+^, (2) Bu_4_N^+^, (3) (MOEOE)_3_MeN^+^, (4) Bu_4_P^+^, and (5) Bu_3_(MOE). Reproduce from reference [93].

**Figure 20:**
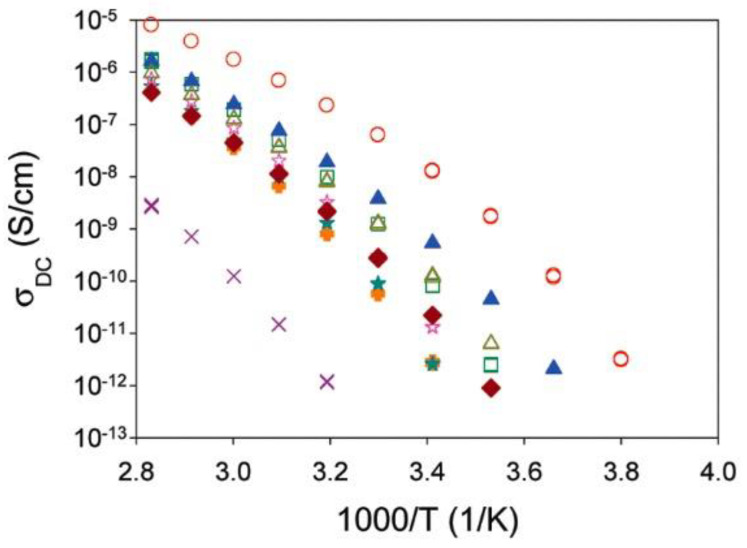
The influence of different counter cations on the ion conductivity (purple x is Na^+^, orange circle solid is Me_4_N^+^; cyan solid star is BuMeIm^+^; pink open star is (MOE)MeIm^+^, dark red solid diamond is Bu_4_N^+^, blue solid triangle is Bu_4_P^+^, olive green triangle is Me_3_(MOE)P^+^, red open circle is (MOEOE)_3_MeN^+^. Reproduced from reference [93].

Ion conductivities significantly improved as a result of the substitution of Na^+^ (which has a high electrostatic interaction) for quaternary cations at 30 °C, as illustrated in Figure 19 and Figure 20. Comparing the similarity in size and chemical structure between quaternary ammonium cations (Bu_4_N^+^) and phosphonium cations (Bu_4_P^+^), phosphonium-based PIL showed a significant decrease in *T*_g_ from 13 °C to 0 °C.

**Figure 21:**
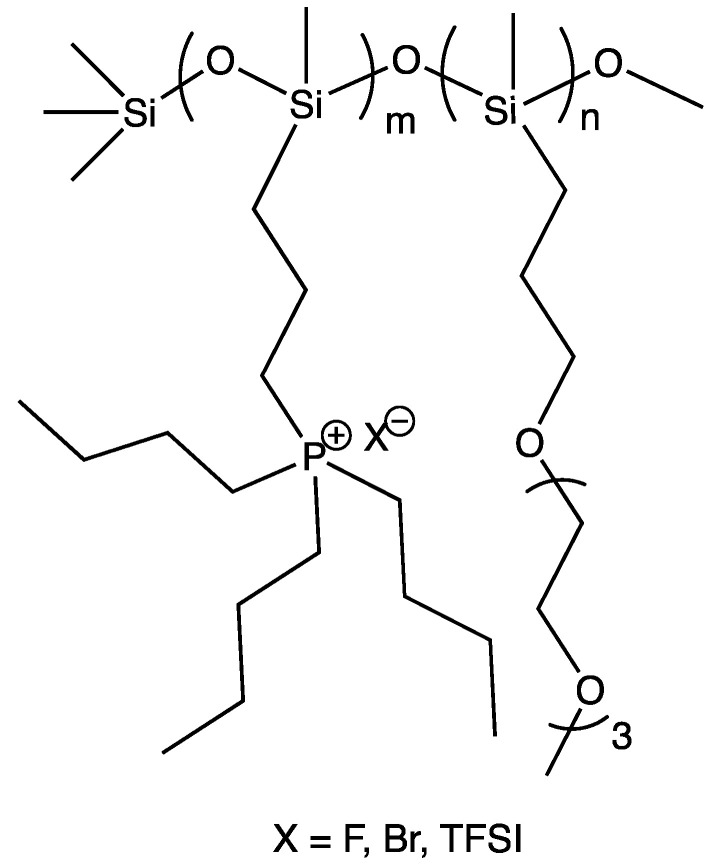
The structure of phosphonium siloxane ionomers. Reproduced from reference [114].

Due to weaker physical crosslinking, better mobility (lower *T*_g_) was made possible by phosphonium-based PILs’ weaker electrostatic interactions. In contrast to the findings of Colby and colleagues, Long et al. showed no *T*_g_ change between nitrogen-based and phosphonium-based PILs, as was previously addressed [27]. This ambiguous observation is clarified by the molar volume of pendant groups [114,115]. Colby et al. stated that the increased side group volume, including counterions, reduces *T*_g_ until the bigger molar volume limit [116]. After this point, the molar volume of side groups has little effect on *T*_g_. For the phosphonium siloxane ionomers (Figure 21) studied in this research, it is noted that at ion contents of 11 mol% or lower, the siloxane ionomers with TFSI counterion exhibit similar *T*_g_s but superior conductivity to the ionomers containing Br^−^ or F^−^ anions (as shown in Table 5). The phosphonium ionomers have lower *T_g_s* than normal ionomers with C-C backbones because their backbone is polysiloxane, a very flexible polymer chain.

Additionally, the Tf_2_N counter-anions in the studies by Long et al. [111] were bulky enough to surpass the higher molar volume limit, rendering polymers resistant to additional size changes. Quaternary cations and carboxylate anions are not as bulky as Tf_2_N counter anions in Colby et al.’s work [93]. Thus, the larger *T*_g_ fluctuations were probably induced by ion size variation. After an ion exchange from Cl to Tf_2_N counter anions demonstrated by Long et al., the *T*_g_ of phosphonium-containing copolymers was reduced over 100 °C [117]. Another factor to consider is a thorough analysis of experimentally determined data, such as *T*_g_ and ionic conductivity. To evaluate the AC conductivity in this situation, Long et al. used impedance spectroscopy, while Colby et al. used a broadband dielectric spectrometer. Consequently, the instrumental difference could be the cause of any potential contradictions.

**Scheme 4:**
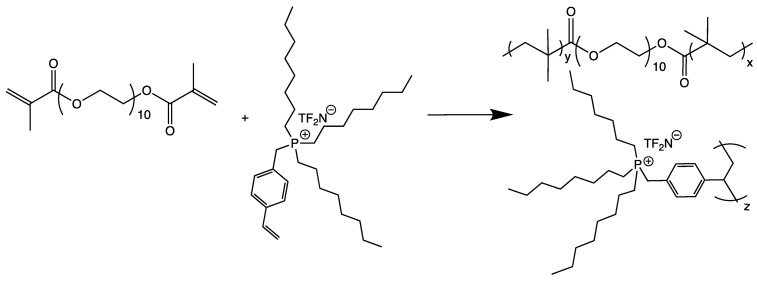
Synthesis of Poly(PEGDMA-co Tri(octyl)phenyl phosphonium Tf_2_N). Reproduced from reference [118].

In the other studies, Long et al. studied the crosslinked PEG matrices in polymer backbones (Scheme 4), where the segmental mobility of the polymer backbone was constrained and exhibited ion conductivity due to the high ion concentrations [118]. Tri(octyl)phenyl phosphonium cations and the bulky Tf_2_N counterions mostly determined *T*_g_, and increasing ion concentration actually caused a decrease in *T*_g_. As a result, larger ion concentrations resulted in improved ion conductivity. The optimum conductivity value was observed at 9 × 10^−2^ S cm^−1^ at ambient temperature.

Briefly, counterion exchange is a reliable method to enhance PIL ion conductivity. The electrostatic interactions are tuned in addition to plasticization to achieve higher conductivity. Larger cations typically act as plasticizers, lowering *T*_g_ because ion interactions have weaker Coulombic forces, which act as physical crosslinks. Additionally, bulky counterions introduce more free volume and achieve lower *T*_g_s, which facilitate mass transfer (ion conductivity).

### 4.5. Sability of Phosphonium PILs

The stability of a polymer ionic liquid (PIL) after synthesis can be influenced by various factors, including its chemical structure, processing conditions, and environmental factors. PIL should be stable at the desired operating temperatures. Basically, phosphonium-based ionic liquids (ILs) are stable in high-temperature applications due to their non-flammable properties. The influence of counterion on the thermal properties of phosphonium polyelectrolytes was also reported that anion exchange from Cl^−^ to BF_4_^−^, TfO^−^, and Tf_2_N^−^ improves the thermal stability (>400 °C).

The stability of the PIL in different solvents, moisture, or the presence of specific chemicals should be considered [119]. PILs may be susceptible to degradation or swelling when exposed to certain solvents or chemicals. For example, polar protic and polar aprotic solvents can influence the conductivity of imidazole-based PIL membranes [120]. The ion conductivity of PIL films in propylene carbonate was three times smaller than that in a polar protic solvent. Generally, the chemical stability of the PIL is important to ensure that it does not undergo unwanted reactions or degradation over time. Factors such as oxidation, hydrolysis, or exposure to specific chemicals can impact stability. There are two common reactions, the first is deprotonation and the second electron transfer, cause the stability of phosphonium ionic liquids under basic conditions [121,122]. Bases, such as hydroxide, can react with the phosphonium ions and may cause the decomposition of the phosphonium ionic liquids. PILs have a tendency to absorb moisture from the surrounding environment, which can affect their stability and performance [123]. The presence of moisture can cause swelling of the PIL and result in changes in its physical properties, such as increased volume or altered mechanical properties. This can influence the mobility of ions within the PIL matrix and ion conductivity. Phosphonium-based ionic liquids are relatively stable under radiolytic degradation [124], but radiolytic dissociation of the P–C bond in the cation units results in the formation of small organic species over time. The formation of these byproducts results in changes in ion conductivities. Similarly, its polymers are expected to show the same behavior.

## 5. Conclusions

ILs, base polymer electrolytes, and PILs are possible good candidates for electrolytes for next-generation batteries because of their ionic conductivity and thermodynamic stability. In lithium battery electrolytes, lithium salt mixtures with ILs offer good electrochemical stability and lessen electrolyte decomposition, increasing cycle lifetimes. Improved safety and stability at high temperatures are also provided by the non-flammability and low volatility characteristics of ILs. Engineering compositions with lower viscosities is an important development objective because the relatively high viscosities of ILs appear to be impeding their commercial use. Additionally, it is still unclear how lithium ions are transported and solvated in ILs, which can be confusing when designing ILs for electrolyte applications. Therefore, more investigation into the solvation and transport of lithium salts at the molecular and ionic scales in ILs PIL-based materials chemistry has entered a highly interesting period because of the continual discovery of new PIL chemical structures. Chemists have made progress in the synthesis and structural characterization of new PILs, and further work will be done to integrate PILs into usable materials that address problems and difficulties in areas such as life science, energy, and the environment.

In this review, we described the differences between two types of PILs, namely nitrogen-based and phosphonium-based ones. The cation structure differs dramatically depending on whether the cationic atom is nitrogen or phosphorus due to differences in atomic radius and electronegativity. Clearly, phosphonium ILs have some advantages over other IL types, such as greater thermal stability, lower viscosity, and greater stability in strongly basic or strongly reducing environments. The increased thermal stability of phosphonium polyelectrolytes is a result of their resistance to Hofmann elimination. It is shown that phosphonium-based PILs have an advantage regarding electrical and thermal properties compared to nitrogen-based ones. Phosphonium-based PILs, on the other hand, have intriguing potential as solid/gel state electrolytes, such as ion gels. Hence, phosphonium-based PILs with microphase-separated morphologies, programmable and responsive mechanical properties, and continuous ion conductivity improvement have a promising future in innovative electrolyte applications.

### Future Recommendation

Naturally, it is preferable to create PILs with a large, specific surface area. The best nanostructures for chemical accessibility or absorption purposes are porous (especially mesoporous) ones. Due to the defined cross-linking sites between the polymer chains, polymerization can produce an ordered and well-defined interior structure in addition to the external morphology of PILs. On the other hand, decreasing the PIL’s individual particle size to the nanoscale and eventually attaining the size of a single polymer chain would be of particular interest.

As mentioned before, the potential advantages of phosphonium-based polymer electrolytes against ionic liquids are their thermal and chemical stability, as well as their mechanical improvement to the matrix. Thus, the development of shape memory recovery after scratching and impact can be one of the important fields of future study.

Computational modeling and simulation can help investigate the structure-property relationship [125,126,127,128]. Basically, a fundamental understanding of factors affecting conductivity such as polymer matrix and lithium salt interaction, humidity, electrode/electrolyte interfaces, and morphology of the matrix might be correlated with the experimental and computational studies.

Previously, a study was conducted within the scope of the effect of molecular weights of polymers containing ammonium-based salts on conductivity [101]. It may be useful to examine the conductivity of polymers with well-designed molecular weight and distribution by controlled polymerization techniques and to examine the factors such as salt type, backbone flexibility, rigidity, and hydrophobicity affecting conductivity.

Additionally, physical, electrical, thermal, manufacturing defects, and even battery age can all contribute to or exacerbate the thermal risks of lithium-ion batteries (LIBs). Due to their activity and combustibility, traditional battery components often pose a significant thermal hazard. Additionally, under abusive conditions, a number of side reactions involving electrodes and electrolytes may take place, which would further contribute to the thermal failure of LIBs. The use of safety devices, including fire-retardant additives, was discussed in order to lessen these risks. Thus, phosphorus-based polymer compounds are important candidates as they are inherently flame-retardant, do not release into the environment, and provide flame retardant properties for a long time. Phosphorus compounds increase the thermal stability of the end products via gas phase and/or solid phase actions. During combustion, phosphorus not only affects the combustion mechanism by increasing the amount of charcoal residue in the matrix, forming a pyrophosphonate layer to prevent the flame from reaching the inside, but also exhibits flame retardant properties by inhibiting the flame in the gas phase via radical scavenger.

Besides the phosphonium-based polymeric ionic liquids, black phosphorus and red phosphorus can construct a new single elemental heterostructure that would be beneficial to electron transfer and exhibit superior electrochemical performance. It seems that recovering phosphorus from waste batteries such as lithium will be an important task in the future. Since phosphate rock was listed as a critical raw material by the EU in May 2014 [127], researchers have taken an important step towards sustainability, which is the recovery and recycling of phosphorus from wastewater. It is crucial to investigate all potential sources of phosphorus, including those derived from sewage and human excreta. Although large amounts of energy and reactants are needed to recover phosphorus from sludge or wastewater, the depletion of mineral phosphorus has become a great concern, and the environmental impacts of non-leaching flame retardants are also more important overall. It was also presented that using biobased formulations in polymer matrixes has more significant impacts than their fossil counterparts [128]. Thus, the use of recycled phosphorus in PILs can be explored in the near future.

## Figures and Tables

**Table 1:** Various works of phosphonium-ionic liquid-doped polymer electrolyte.

Doped Phosphonium Ionic Liquid	Polymer Host	Conductivity Value (S cm^−1^)	Electrochemical Performance (V vs. Li^0^/Li^+^)	Ref.
trihexyltetradecylphosphonium bis(trifluoromethylsulfonyl)imide, (P_14,6,6,6_)(Tf_2_N)	Polypropylene glycol (PPO)	0.13 × 10^−3^ at 100 °C	3.95	[69]
trihexyltetradecylphosphonium bis(trifluoromethylsulfonyl)imide, (P_14,6,6,6_)(Tf_2_N)	Polyethylene oxide (PEO)	4.2 ×10^−5^ at ambient	3.34	[70]
trihexyltetradecylphosphonium bis(trifluoromethylsulfonyl)imide, (P_14,6,6,6_)(Tf_2_N)	Poly(vinyl chloride) (PVC)	2.4 × 10^−6^ at ambient	N.A	[79]
trihexyltetradecylphosphonium bis(trifluoromethylsulfonyl) amide (P_14,6,6,6_)(Tf_2_N)	Poly(vinylidenefluoride-*co*-hexafluoropropylene) P(VdF-*co*-HFP)	3.2 × 10^−6^ at ambient	3.4	[80]
trimethylisobutylphosphonium bis(fluorosulfonyl)imide (P_111i4_FSI)	Poly(diallyldimethylammonium) (PDADMA)	0.49 × 10 ^−3^ at 40 °C	5.0	[78]
trihexyltetradecylphosphonium bis(trifluoromethylsulfonyl) amide (P_14,6,6,6_) (Tf_2_N)	Polypyrrole	0.6 at ambient	-	[81]
triphenylphosphonium-butyl sulfonate (PPh_3_.ZIL)/dodecylbenzenesulfonic acid	Polyaniline (PANI)	4.9 at ambient	-	[82]
triethylpentylphosphonium bis(trifluoromethanesulfonyl)amide, (P_2225_) (Tf_2_N))	Tetra-arm poly (ethylene glycol) (TetraPEG)	2.78 × 10^−1^ at ambient	4.2	[77]
trihexyltetradecylphosphonium dicyanamide (P_14,6,6,6_)(DCA)	Diglycidyl ether of bisphenol A (DGEBA)-based epoxy	~10^−6^ at room temperature	-	[83]
[P_4441_][Tf_2_N]	Polymethylmethacrylate (PMMA)	7.94 × 10^−5^ at 30 °C	-	[84]
[P_2225_][Tf_2_N]	Polymethylmethacrylate (PMMA)	1.46 × 10^−4^ at 30 °C	-	[84]

**Table 2:** Conductivity values of various phosphonium PILs.

Used Phosphonium Polymeric Ionic Liquid	Polymer Matrix	Conductivity (S cm^−1)^	Ref.
PAMPS HP^+^_444_	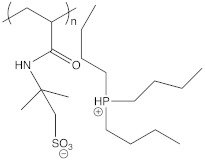	5 × 10^−6^ at 30 °C	[97]
[P(N(Me)Cy)_4_]^+^ polyethylene	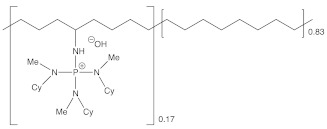	2.2 ×10^−2^ at 22 °C	[29]
[Bu_4_P^+^]polyurethane	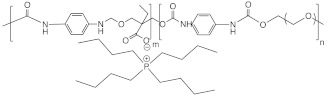	~10^−6^ at 100 °C	[93]
[Me_3_(MOE)P^+^] polyurethane	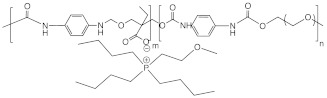	~10^−6^ at 100 °C	[93]
1,4bis(di-phenyl phosphine) butane 1,12 di-trifluoro sulfonyl imide dodecane	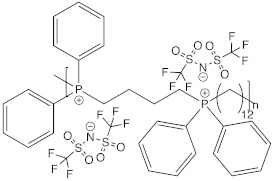	7 × 10^−6^	[98]
PEO-grafted Siloxane Phosphonium Ionomers	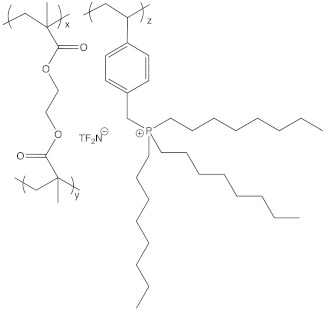	9 × 10^−2^ at ambient temperature.	[99]

**Table 3:** Comparison between phosphonium-based PILs and ammonium-based PILs [111].

PIL	T_d_ (°C)	*T*_g_ (°C)	Conductivity (S cm^−1^)
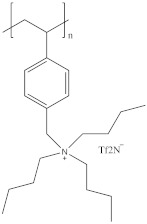	330	62	5.01 × 10^−5^
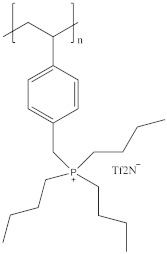	437	66	1.6 × 10^−4^

**Table 4:** Charge distribution of Bu_4_P^+^, Bu_4_N^+^, and BuMeIm^+^ from ab initio calculation [93].

	Center (P or N)	α—CH_2_	β—CH_2_
Bu_4_P^+^ 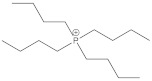	1.1	−0.2	0
Bu_4_N^+^ 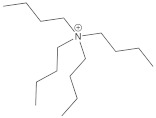	−0.5	0.3	0
BuMeIm^+^ 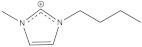	−0.3, −0.3	0.4	0

**Table 5:** Glass transition temperature and conductivity value of phosphonium siloxane ionomers with different anions [114].

Samples	Anion	*n*	*T*_g_ (°C)	Conductivity at 30 °C (S cm^−1^)
PSPE-5Br(3)	Br^−^	5	−83	5.60 × 10^−7^
PSPE-8Br(3)		8	−82	7.50 × 10^−7^
PSPE-11Br(3)		11	−80	6.88 × 10^−7^
PSPE-22Br(3)		22	−86	1.44 × 10^−6^
PSPE-5TFSI(3)	TFSI^−^	5	−81	1.09 × 10^−5^
PSPE-8TFSI(3)		8	−81	3.12 × 10^−5^
PSPE-11TFSI(3)		11	−80	2.12 × 10^−5^
PSPE-5F(3)	F^−^	5	−80	1.9 × 10^−7^
PSPE-8F(3)		8	−83	2.00 × 10^−7^
PSPE-11F(3)		11	−82	1.7 × 10^−7^
PSPE-22F(3)		22	−73	7.4 × 10^−7^

## Data Availability

Not applicable.
